# Nuclear Factor Kappa B Promotes Ferritin Heavy Chain Expression in *Bombyx mori* in Response to *B. mori* Nucleopolyhedrovirus Infection

**DOI:** 10.3390/ijms231810380

**Published:** 2022-09-08

**Authors:** Linbao Zhu, Yingxue Liu, Ancheng Wang, Xiya Chen, Handan Zhu, Zhihao Huang, Huihua Cao, Shihuo Liu, Jiaping Xu

**Affiliations:** 1 School of Life Sciences, Anhui Agricultural University, Hefei 230036, China; 2 Anhui International Joint Research and Developmental Center of Sericulture Resources Utilization, Hefei 230036, China

**Keywords:** BmNPV, BmFerHCH, BmRelish, NF-κB cis-regulatory elements

## Abstract

Ferritin heavy chain (FerHCH) is a major component of ferritin and plays an important role in maintaining iron homeostasis and redox equilibrium. Our previous studies have demonstrated that the *Bombyx mori* ferritin heavy chain homolog (BmFerHCH) could respond to *B. mori* nucleopolyhedrovirus (BmNPV) infection. However, the mechanism by which BmNPV regulates the expression of BmFerHCH remains unclear. In this study, BmFerHCH increased after BmNPV infection and BmNPV infection enhanced nuclear factor kappa B (NF-κB) activity in BmN cells. An NF-κB inhibitor (PDTC) reduced the expression of the virus-induced BmFerHCH in BmN cells, and overexpression of BmRelish (NF-κB) increased the expression of virus-induced BmFerHCH in BmN cells. Furthermore, BmNPV infection enhanced BmFerHCH promoter activity. The potential NF-κB cis-regulatory elements (CREs) in the BmFerHCH promoter were screened by using the JASPAR CORE database, and two effective NF-κB CREs were identified using a dual luciferase reporting system and electrophoretic mobility shift assay (EMSA). BmRelish (NF-κB) bound to NF-κB CREs and promoted the transcription of BmFerHCH. Taken together, BmNPV promotes activation of BmRelish (NF-κB), and activated BmRelish (NF-κB) binds to NF-κB CREs of BmFerHCH promoter to enhance BmFerHCH expression. Our study provides a foundation for future research on the function of BmFerHCH in BmNPV infection.

## 1. Introduction

*Bombyx mori* is an economically important insect and serves as a model organism of lepidopterans for studying insect resistance and immunity [1]. *B. mori* nucleopolyhedrovirus (BmNPV) is a serious pathogenic virus that specifically infects domestic silkworms, resulting in enormous losses for the sericulture industry. BmNPV is a double-stranded DNA baculovirus with two virion phenotypes: obliterator viruses (ODVs) and bud viruses (BVs). ODVs are embedded in a large number of polyhedrons and remain active in harsh environments, facilitating virus transmission between hosts. BVs are responsible for systemic infection in silkworms [2]. There is currently no effective treatment available for BmNPV infection [3].

Ferritin is a complex protein composed of heavy chain homologue (FerHCH) and light chain homologue (FerLCH) and plays an important role in maintaining iron homeostasis [4,5]. FerHCH contains a ferroxidase centre, which consumes oxygen or hydrogen peroxide to catalyse the oxidation of Fe^2+^ to Fe^3+^ and has important iron chelation and antioxidant functions. FerLCH provides auxiliary functions [6,7]. Ferritin expression is influenced by many factors, including free iron ions, oxidants and pathogenic stimuli [8]. Iron is the predominant factor regulating ferritin synthesis at the transcriptional level, affecting the binding ability of iron-regulated proteins (IRP) to bind to iron response elements (IREs) at the 5′ untranslated region (UTR) of ferritin messenger RNA (mRNA) [9]. Further, under oxidative pressure, Nrf2 and JunD can bind to antioxidant response elements (ARE) and regulate *FerHCH* transcription [10,11], Research in Rana sylvatica indicates that the activation of nuclear factor kappa B (NF-κB) promotes FerHCH transcription in response to oxidative stress during freezing and thawing [12]. It was worth mentioning that a high FerHCH expression has been reported in numerous viral infections. The hepatitis C virus could upregulate host FerHCH to promote HCV infection [13]. The viral protein Nef mediates NF-κB activation to induce ferritin secretion in human immunodeficiency virus type 1 (HIV-1) infection [14]. After infection of human cells with dengue virus (DENV) and East respiratory syndrome coronavirus (MERS-CoV), and during infection of rat cells with Theiler’s murine encephalomyelitis virus (TMEV), host cells may release multiple inflammatory cytokines, including tumour necrosis factor alpha (TNF-α) and interleukin 1 beta (IL-1β) [15,16,17]. TNF-α and IL-1 are well-known regulators of FerHCH expression [18,19], and TNF-α regulates FerHCH mRNA expression via NF-κB activation [20].

NF-κB is a ubiquitous transcription factor, and activated NF-κB regulates the transcription of various immune-related genes [21]. In insects, there are two central immune signalling cascades for activating NF-κB: the Toll and immune-deficient (IMD) pathways. In the Toll pathway, cactus is an inhibitor of Dif/Dorsal (NF-κB), and once cactus is degraded, Dif/Dorsal (NF-κB) is transferred from the cytoplasm to the nucleus to regulate downstream genes [22,23,24]. In the IMD pathway, the ANK region of the Relish protein (NF-κB) is excised, resulting in Relish-FL being converted to Relish-act, which is transported to the nucleus and performs its function [25]. Numerous clinical studies have demonstrated that NF-κB activation is associated with viral infection, such as hepatitis C virus, Epstein–Barr virus, and African swine fever virus [26,27,28,29]. According to a previous study, *Antheraea pernyi* nuclear polyhedrosis virus induces proinflammatory cytokines and nitric oxide (NO) production through mitogen-activated protein kinase (MAPK) and NF-κB pathways [30]. A study on *B. mori* cytoplasmic polyhedrosis (BmCPV) virus demonstrated that the viral peptide vSP27 encoded by BmCPV circRNA could activate the ROS-NF-κB pathway [31].

In our previous research, we found that *BmFerHCH* expression is upregulated in haemolymph during BmNPV infection [32]; however, the mechanism by which BmNPV regulates *BmFerHCH* expression is not clear. Further, according to our previous proteomics data, BmNPV upregulated the expression of BmRelish (NF-κB) in the midgut of two different strains of *B. mori* (Table S1). The present study was conducted to determine whether *BmFerHCH* expression is regulated by NF-κB during BmNPV infection. First, we measured *BmFerHCH* expression and NF-κB activation after BmNPV infection. To understand the role of NF-κB in regulating *BmFerHCH* expression, we analysed its expression after BmNPV infection while inhibiting or enhancing NF-κB activation. Subsequently, we analysed NF-κB cis-regulatory elements (CREs) of *BmFerHCH* by using a dual-luciferase reporter system and electrophoretic mobility shift assay (EMSA). Our exploration of the regulatory mechanism of *BmFerHCH* expression during virus infection provides new insight into the innate silkworm antiviral immune mechanism.

## 2. Results

### 2.1. BmNPV Induced BmFerHCH Expression in BmN Cells

**Figure 1:**
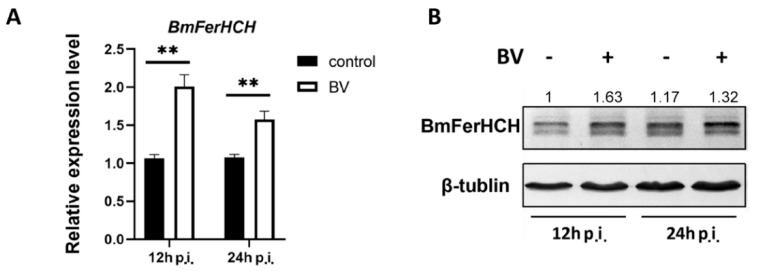
BmFerHCH expression in BmN cells after BmNPV infection. (**A**) RT-qPCR analysis of BmFerHCH expression in BmN cells following BmNPV infection. (**B**) BmFerHCH protein expression in BmN cells following BmNPV infection in BmN cells, using anti-BmFerHCH serum. The numbers represent the ratio of BmFerHCH to β-tubulin after grayscale analysis. The data are presented as the mean ± standard deviation of three independent assays. ** *p* < 0.01.

We analysed BmFerHCH transcription and translation in BmN cells with RT-qPCR and western blotting, respectively, to determine whether BmFerHCH responds to BmNPV infection. BmFerHCH expression increased significantly from 12 to 24 h post-injection (hpi) compared with the control groups (Figure 1). These results suggest that BmNPV may be responsible for inducing BmFerHCH expression.

### 2.2. BmNPV Infection Activated NF-κB

**Figure 2:**
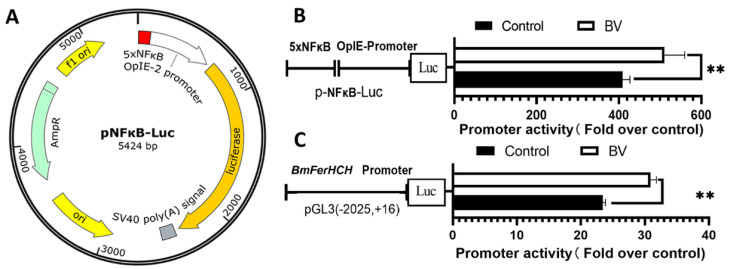
BmNPV activated NF-κB and enhanced BmFerHCH promoter activity in BmN Cells. (**A**) The structure of the reporter plasmid pNF-κB-Luc with the NF-κB response element is shown. Abbreviations: 5×NF-κB, five tandem NF-κB consensus sequences; OpIE 2: constitutive promoter for insect cell expression. (**B**) Twenty-four hours after co-transfection of pNF-κB-Luc and pAc5.1-Renilla, BmN cells were infected with BV and luciferase activity was detected 12 hpi. (**C**) Twenty-four hours after co-transfection of pGL3(−2025, +16) and pAc5.1-Renilla, BmN cells were infected with BV and luciferase activity was detected 12 hpi. The data are presented as the mean ± standard deviation of three independent assays. ** *p* < 0.01.

We constructed the NF-κB reporter gene plasmid pNF-κB-luc to detect NF-κB signal and to evaluate the role of NF-κB during BmNPV infection. It contains a variety of NF-κB binding sites. NF-κB activity strongly correlated with luciferase expression (Figure 2A). Co-transfection of pNF-κB-Luc and pAc5.1-Renilla (reference plasmid stably expressing Renilla luciferase) allowed measuring NF-κB activation in BV-infected BmN cells. The relative luciferase activity in BV-infected BmN cells increased significantly compared with the control group, indicating activation of NF-κB by BV infection (Figure 2B). Furthermore, BmFerHCH promoter activity increased significantly in BV-infected cells (Figure 2C). We speculate that BmNPV infection could activate NF-κB, which could increase BmFerHCH promoter activity.

### 2.3. Inhibiting NF-κB Activation Suppressed BmFerHCH Expression Induced by BV

**Figure 3:**
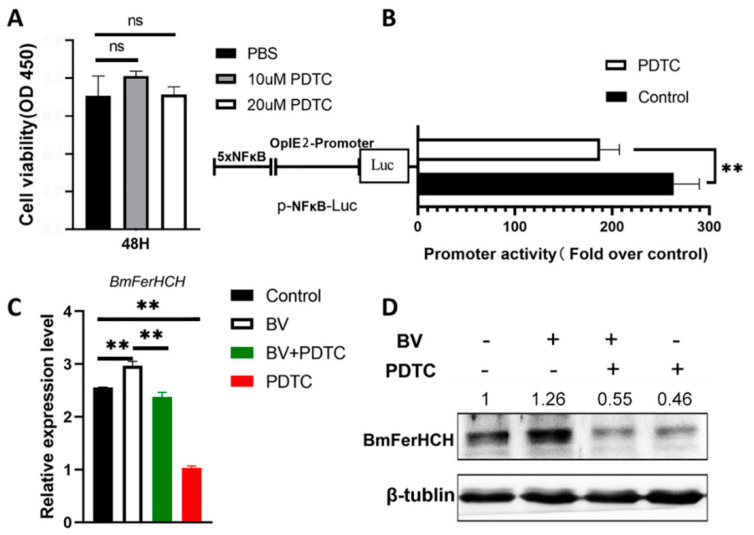
BmNPV-induced BmFerHCH expression was related to NF-κB activation. (**A**) Cell viability after PDTC treatment. (**B**) Inhibitory effect of PDTC on NF-κB; 24 h after co-transfection of pNF-κB-Luc and pAc5.1-Renilla, the relative luciferase activity of the cells was detected after 12 h PDTC treatment (based on the firefly/Renilla luciferase activity ratio). BmN cells were mock infected or infected with BV in the absence or presence of PDTC (20 μM) for 12 h, and BmFerHCH mRNA (**C**) and protein (**D**) expression were determined. BmFerHCH protein was identified by anti-BmFerHCH serum. The numbers on the western blotting represent the ratio of BmFerHCH to β-tubulin after grayscale analysis. The data are presented as the mean ± standard deviation of three independent assays. ** *p* < 0.01.

To investigate whether *BmFerHCH* upregulation induced by BmNPV infection might be due to NF-κB activation, we treated the BmN cells with the NF-κB inhibitor PDTC and determined NF-κB activation by using the dual luciferase reporter gene system. As shown in Figure 3A, cell proliferation was not affected significantly after treatment with 20 μM PDTC for 48 h. We used the pNF-κB-Luc reporter plasmid to detect the effect of PDTC treatment on NF-κB activity in BmN cells. PDTC treatment significantly reduced luciferase signalling in BmN cells compared with the control group (Figure 3B), indicating that PDTC could inhibit NF-κB activation in BmN cells. Next, we measured BmFerHCH mRNA and protein in BmN cells pre-treated with PDTC 12 hpi. Blocking NF-κB activation significantly downregulated BV-induced *BmFerHCH* expression. Further, PDTC inhibited *BmFerHCH* expression in uninfected cells (Figure 3C,D).

### 2.4. Enhancing BmRelish (NF-κB) Activation Promoted BmFerHCH Expression Induced by BV

**Figure 4:**
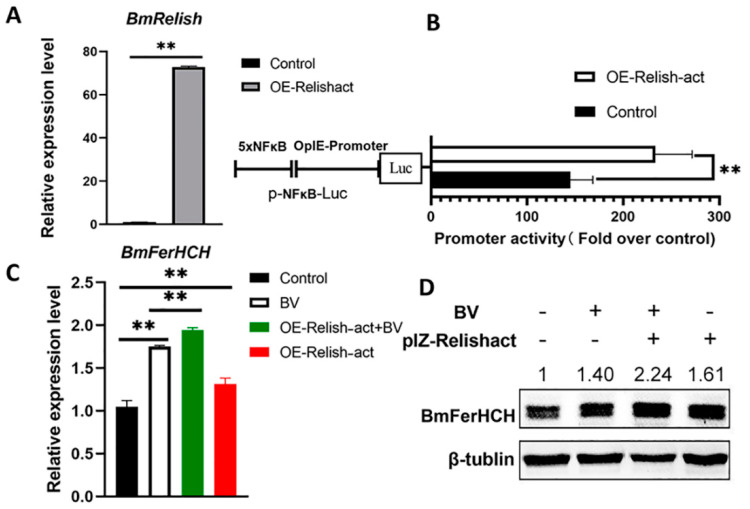
Overexpression of BmRelish-act enhanced NF-κB expression and promoted BmNPV-induced BmFerHCH mRNA and protein expression. (**A**) Overexpression of BmRelish-act in BmN cells was confirmed by analysing the transcript level. (**B**) Overexpression of BmRelish-act enhanced NF-κB transcriptional activity in BmN cells. The effects of BmRelish-act overexpression on BmFerHCH expression in infected or uninfected BmN cells were analysed at the (**C**) transcriptional and (**D**) protein levels. BmFerHCH protein was identified by anti-BmFerHCH serum. The numbers on the western blotting represent the ratio of BmFerHCH to β-tubulin after grayscale analysis. The data are presented as the mean ± standard deviation of three independent assays. ** *p* < 0.01.

BmRelish is a member of the silkworm NF-κB transcription factor family. The complete BmRelish protein forms BmRelish-act after excision of the ANK region via processing. Further, BmRelish-act regulates the expression of various genes [25]. To investigate whether NF-κB positively regulates *BmFerHCH* transcription, we constructed an overexpression vector (pIZ-BmRelish-act) and transfected BmN cells with it. The overexpression efficiency of the BmRelish-act vector is shown in Figure 4A. Luciferase activity analysis revealed that BmRelish-act overexpression significantly increased the pNF-κB-Luc reporter gene compared with the control group (Figure 4B), indicating that BmRelish-act overexpression could enhance NF-κB activation in BmN cells. In addition, we analysed BmFerHCH mRNA and protein expression in BmN cells overexpressing BmRelish-act 12 hpi. BmRelish-act overexpression significantly upregulated BmFerHCH mRNA and protein induced by BV (Figure 4C,D). Combined with the results of NF-κB inhibitor results, we speculate that BmNPV upregulates *BmFerHCH* expression by activating NF-κB.

### 2.5. Enhancing BmRelish (NF-κB) Activation Promoted BmFerHCH Expression Induced by BV

**Figure 5:**
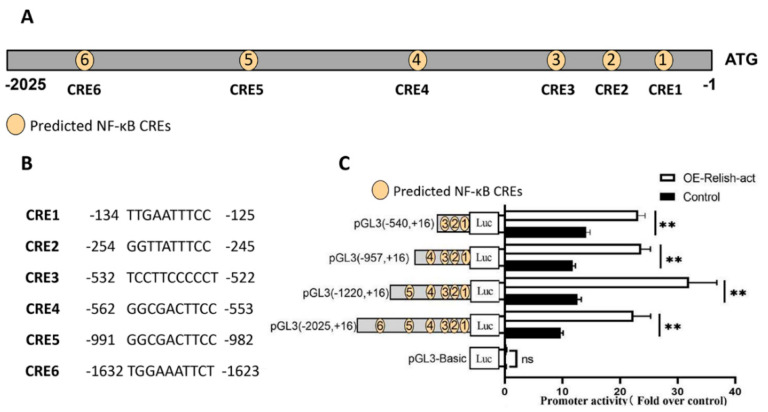
BmFerHCH promoter response to NF-κB signals. (**A**) Potential NF-κB CREs in BmFerHCH promoters. (**B**) Deoxyribonucleotide sequence of NF-κB CREs. (**C**) Determination of luciferase activity of progressively decreasing promoters and their response to BmRelish-act. Yellow circles represent the predicted NF-κB CREs. The data are presented as the mean ± standard deviation of three independent assays. ** *p* < 0.01.

We screened potential NF-κB CREs in the *BmFerHCH* promoter by comparing the NF-κB sequences of multiple species in the JASPAR CORE database accessed on 1 January 2020 (https://jaspar.genereg.net/). This endeavour revealed the presence of six hypothetical NF-κB CREs: CRE1, −134/−125; CRE2, −254/−245; CRE3, −532/−522; CRE4, −562/−553; CRE5, −991/−982; and CRE6, −1632/−1623 (Figure 5A,B). We constructed a series of luciferase reporter vectors containing *BmFerHCH* promoters of different lengths to determine which NF-κB CREs regulate transcription. We transfected BmN cells with each NF-κB-CRE pGL3 vector and pIZ-BmRelish-act. We measured the luciferase activity 48 h after transfection using a dual-luciferase reporter system. The promoter of *BmFerHCH* could respond to BmRelish-act. However, the gradual reduction of NF-κB CREs did not affect the *BmFerHCH* promoter response to BmRelish-act in the first rounds of progressive promoter deletions. These data indicate that NF-κB CREs may be close to the transcription initiation site or contain more than one efficient NF-κB CRE in the *BmFerHCH* promoter (Figure 5C).

**Figure 6:**
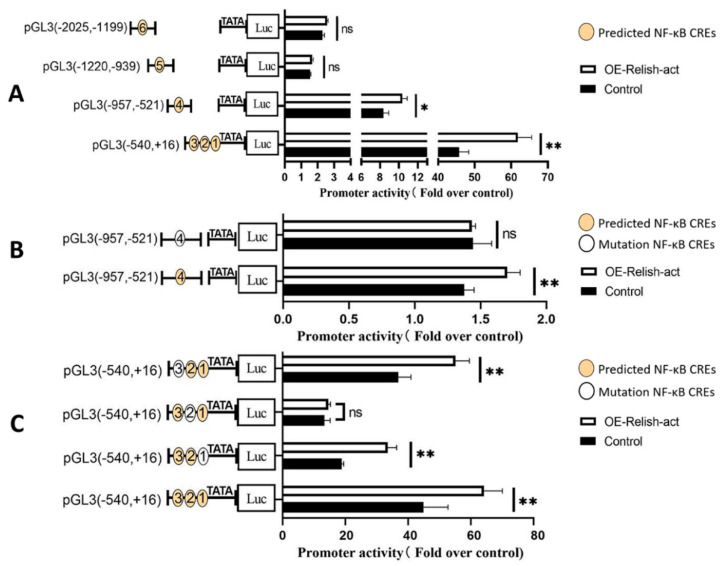
Determination of the luciferase (Luc) activity of mutant *BmFerHCH* promoter. (**A**) The activity of *BmFerHCH* promoters containing different NF-κB CREs and the response to BmRelish-act nucleotide −957 and −521 were detected by dual luciferase activity. (**B**) The promoter activity and the ability to respond to BmRelish-act after CRE4 mutations between nucleotide −957 and-521 were detected by dual luciferase activity. (**C**) The promoter activity and the ability to respond to BmRelish-act after CRE1, CRE2 or CRE3 mutations between nucleotide −540 and +16 were detected by dual luciferase activity. Yellow circles represent predicted NF-κB CREs and white circles represent mutated NF-κB CREs. The data are presented as the mean ± standard deviation of three independent assays. * *p* < 0.05; ** *p* < 0.01; ns indicates no significant difference.

Subsequently, we linked individual wild-type or mutated CREs to luciferase reporter vectors containing core promoters and measured their response to NF-κB based on luciferase activity. BmRelish-act significantly increased the BmFerHCH promoter transcriptional activity through CRE1–CRE3 and CRE 4, and the promoter region containing CRE1–CRE3 had the highest transcriptional activity (Figure 6A), suggesting that CRE1–CRE3 and CRE4 are active. To confirm the BmFerHCH promoter contains the NF-κB CREs, we mutated CRE1, CRE2, CRE3 and CRE4 and determined the activity of the mutant promoter. When CRE2 and CRE4 were mutated, the BmFerHCH promoter did not respond to BmRelish-act (Figure 6B,C). Hence, we believe that the CRE2 and CRE4 in the BmFerHCH promoter region are NF-κB CREs.

### 2.6. BmRelish Binds to NF-κB CRE2 and CRE4 in the BmFerHCH Promoter

**Figure 7:**
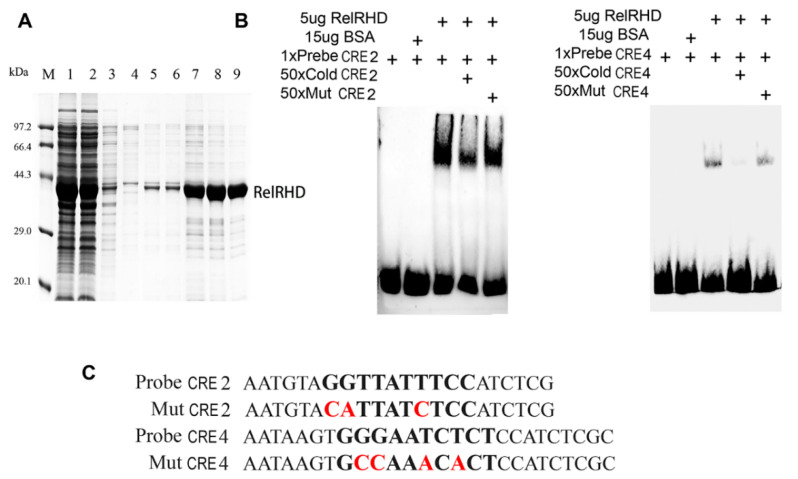
Expression and purification of recombinant RHD domain of BmRelish and EMSA of purified RHD domain binding with CRE 2 and CRE 4. (**A**) Expression and purification of recombinant RHD; the predicted molecular weight is 39.3 kDa. Lane identities—1: supernatant of cell lysate; 3–6: wash solution containing 15, 30, 60 and 120 mM imidazole, respectively; 7–9: eluent buffer containing 250 mM imidazole. (**B**) EMSA showing the binding ability of recombinant RHD to NF-κB CRE2 and CRE4. Cold CRE: probes that are not labelled with biotin; mutant CRE: the mutated probe; BSA: bull serum albumin. (**C**) The wild/cold probe sequence and mutated probe sequence. The sequence in bold represents the core sequence of the CRE and mutated sequences are red.

We expressed and purified the recombinant RHD domain of BmRelish to determine whether BmRelish could bind to NF-κB CRE2 and CRE 4 in the BmFerHCH promoter (Figure 7A). The wild-type and mutated probe sequences are shown in Figure 7C. EMSA revealed that the recombinant RelRHD protein could bind to biotin-labelled probes of CRE2 and CRE4. The binding bands were weakened after the addition of unlabelled CRE2 and CRE4 probes. In contrast, the addition of mutated probes did not affect the binding bands (Figure 7B). These results indicate that CRE2/CRE4 probes interact specifically with the RHD domain of BmRelish.

## 3. Discussion

BmNPV is a major pathogen of *B. mori*, causing severe losses to the sericulture industry each year. In recent years, numerous genes and proteins, including ferritin, have been found to be involved in BmNPV infection [32]. Our laboratory has demonstrated that *BmFerHCH* expression is upregulated in the haemolymph after ODV feeding infection, and the natural ferritin complex extracted from haemolymph interacts directly with BmNPV [32]. In the present study, we found that BmFerHCH mRNA and protein expression was induced by BmNPV infection of BmN cells (Figure 1). In recent years, researchers have found that ferritin is involved in viral infections. For example, HCV inhibits apolipoprotein B100 expression by upregulating host FerHCH, changes that are beneficial to viral replication [13]. After *Procambarus clarkii* is attacked by the white spot syndrome virus, ferritin mRNA and protein expression increase significantly in blood cells and the hepato-pancreas [33]. Therefore, we consider that BmNPV could induce BmFerHCH expression.

BmRelish is a transcription factor that is part of the *B. mori* NF-κB family. Activated BmRelish can enter the nucleus and bind to gene promoters to regulate gene transcription [34]. By analysing previous proteomics data from our lab, we discovered that BmNPV upregulated the expression of BmRelish protein in the midgut of two different strains of *B. mori* (Table S1). In the present study, luciferase activity of NF-κB reporter gene assay showed enhanced NF-κB activation in BV-infected BmN cells (Figure 2B). Consistently, Hua et al. [35] found that BmNPV infection facilitates the conversion of BmRelish-FL to BmRelish-act, leading to increased NF-κB activity in BmE cells. Based on these results, it can be concluded that BmNPV infection enhances NF-κB activity by regulating BmRelish.

BmFerHCH was induced by BmNPV infection, and BmNPV infection enhanced BmRelish (NF-κB) activity in BmN cells. We hypothesise that BmFerHCH expression is regulated by BmRelish (NF-κB) in silkworms. It has been reported that in macrophages, the HIV-1 protein Nef mediates NF-κB activation and induces ferritin secretion [14]. The inhibition of constitutively activated NF-κB suppresses the expression of the heavy ferritin chain in cutaneous T-cell lymphoma [36]. We also evaluated whether BmNPV-induced BmFerHCH expression is related to BmRelish activation. We found that the NF-κB inhibitor PDTC eliminated the ability of the virus to induce BmFerHCH. It significantly inhibited mRNA and protein expression of BmFerHCH in infected and uninfected BmN cells (Figure 3). On the other hand, overexpression of activated BmRelish could significantly enhance NF-κB signalling and promote the expression of BmFerHCH in uninfected and virus-infected BmN cells (Figure 4). These results suggest that BmRelish (NF-κB) plays an essential role in maintaining *BmFerHCH* expression; the virus may regulate BmFerHCH expression by affecting BmRelish (NF-κB) activity. Moreover, *BmFerHCH* promoter activity was significantly increased in BV-infected cells (Figure 2C). Hence, it is necessary to investigate the promoter of *BmFerHCH* to determine whether NF-κB could regulate transcription by affecting the promoter activity.

Eukaryotic gene promoters contain numerous CREs for binding transcription factors; CREs play an important role in gene expression regulation [37,38]. We searched for potential NF-κB CREs in the *BmFerHCH* promoter and investigated their function. A comparison of luciferase activity before and after mutation of a single NF-κB CREs demonstrated that CRE2 and CRE4 are critical elements of the *BmFerHCH* promoter response to BmRelish-act (Figure 6). EMSA indicates that the RHD domain of BmRelish can bind to the NF-κB CRE2 and CRE4 (Figure 7). It is not unusual for a promoter to contain more than one active and similar set of CREs. Zhang et al. [39] studied the *BmIGFLP* gene and discovered that signal transducer and activator of transcription (STAT) CRE1 and CRE3 in the promoter region of *BmIGFLP* have a cumulative effect on transcription. There is increasing evidence that NF-κB-mediated innate immunity plays an important role in bacterial and some viral infections in invertebrates [40]. *E. coli* induces the expression of epidermal protein BmCPT1 through NF-κB, and BmCPT1 participates in immunity by promoting the expression of antimicrobial peptides [22]. Moreover, bacterial or viral infection may significantly increase the transcription of *BmFerHCH* and *BmFerLCH* in silkworm haemolymph [32]. Based on this information and our data, we hypothesise that ferritin expression induced by BmNPV or bacterial infection involves activating NF-κB.

**Figure 8:**
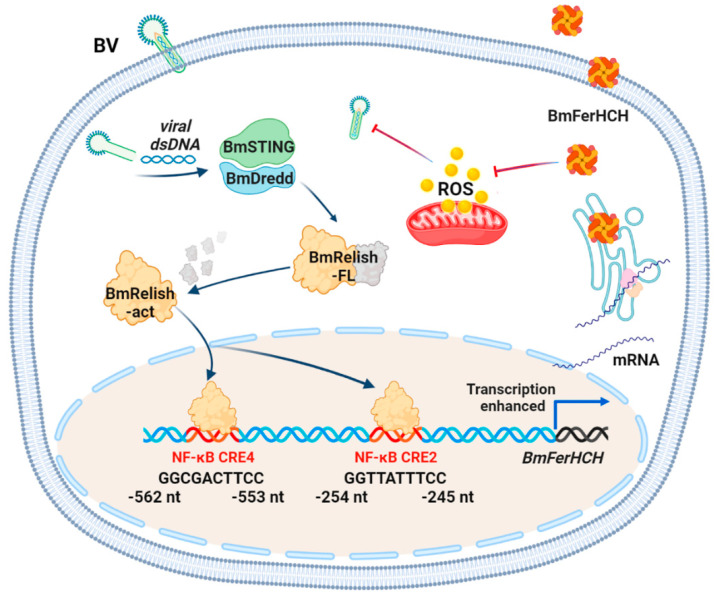
Diagram of the proposed molecular regulation of BmFerHCH expression by BmNPV via BmRelish. After BV invasion, BmRelish/NF-κB is activated by BmSTING-BmDedd. Activated BmRelish binds to the promoter region of BmFerHCH in the nucleus and enhances its transcription, ultimately leading to elevated BmFerHCH protein. On the other hand, BmNPV infection enhances ROS production in the host. High BmFerHCH expression can inhibit ROS, thereby affecting viral replication.

In conclusion, NF-κB activity is essential for maintaining the regular expression of *BmFerHCH* in silkworms. BmNPV infection boosts the NF-κB activity by processing BmRelish; the activated Relish enters the nucleus and binds to NF-κB CRE2/CRE4 to enhance *BmFerHCH* transcription. BmNPV infection could lead to cytopathic effects by enhancing reactive oxygen species (ROS) production, and the increased expressed BmFerHCH could suppress the ROS induced by BmNPV [41,42]. Therefore, we speculate that the expression of BmFerHCH involved in BmNPV infection is dependent on NF-κB and facilitates BmNPV proliferation by inhibiting ROS (Figure 8). These findings should help to elucidate the regulatory pathway of ferritin in NPV invasion and other baculovirus pathogenesis.

## 4. Materials and Methods

### 4.1. Cell Cultures, Virus and Transfection

BmN cell lines were maintained in TC-100 insect medium (AppliChem, Darmstadt, Germany) supplemented with 10% foetal bovine serum (Excell, Shanghai, China). The cells were incubated at 27.5 °C. BmNPV BV was stored in our laboratory. The viral titres were calculated and expressed as 50% tissue culture infectious doses (TCID50) per mL according to Kaerber’s method. Cells were infected with BV at a multiplicity of infection (MOI) of 2. The *Bombyx mori* nucleopolyhedrovirus (BmNPV) genomes accessed on 6 May 2009, taxid:271108 (https://www.ncbi.nlm.nih.gov/).The *B. mori* ovarian cell line (BmN) accessed on 23 September 2021, Accession: CVCL_Z633 (https://web.expasy.org/cellosaurus/).

### 4.2. Protein Expression, Purification and Antibody Preparation

The pET-30a-BmFerHCH and pET-30a-RelRHD plasmids were introduced into *Escherichia coli* BL21 for protein expression. *B. mori* ferritin subunit precursor (GenBank record: NM_001044115) and *B. mori* NF-κB p110 subunit (BmRelish) (GenBank record: XM_038012673) were cloned by polymerase chain reaction (PCR) with primers based on conserved sequences among different species (Table S2) and inserted into the pET-30a plasmid. Following this, the recombinant plasmids were sequenced and transformed into *E. coli* BL21 competent cells (TransGen, Beijing, China). Recombinant protein expression was induced using 1 mM isopropyl-β-thiogalactopyranoside (IPTG) at 37 °C (180 rpm) overnight. After centrifugation at 7500× *g* for 5 min at room temperature, the *E. coli* cells were washed three times using phosphate-buffered saline (PBS, pH 7.4) and lysed by the BeyoLytic™ Bacterial Native Protein Extraction Reagent (Beyotime, Shanghai, China) for 20 min at room temperature. After centrifugation at 12,000× *g* for 10 min at 4 °C. The 6 × His-tagged recombinant protein in the supernatant was bound using High Affinity Ni-NTA Resin (GenScript, Nanjing, China) and washed two times using wash solution (15, 30, 60 and 120 mM imidazole). After that, the protein is eluted using the eluent (250 mM imidazole) and transferred to PBS using dialysis membranes. The molecular weight of the purified protein was analysed by 12% sodium dodecyl sulfate-polyacrylamide gel electrophoresis (SDS-PAGE) followed by staining with Coomassie Brilliant Blue R250, and purity of the protein was analysed using imagej software; purity greater than 85% was considered eligible. Rabbit-derived antiserum against BmFerHCH (anti-BmFerHCH-serum) was prepared by HuaAn Biotechnology Ltd. (Hangzhou, China).

### 4.3. Western Blotting

A total of 1 × 10^6^ cells were collected and washed three times with precooled phosphate-buffered saline (PBS). In total, 300 μL of Cell Lysis Buffer for Western and IP (Beyotime, Shanghai, China) was added to the cells. They were incubated for 20 min and then centrifuged at 4 °C at 12,000 rpm for 10 min; the supernatant containing protein was removed. The protein concentration of the sample was calculated according to standard curve analysis. An appropriate amount of 5× sodium dodecyl sulphate (SDS) loading buffer was added and heated for 5 min at 100 °C. Total protein was isolated by 12% SDS–polyacrylamide gel electrophoresis. After electrophoresis, the protein was transferred to polyvinylidene difluoride (PVDF) membrane with a semi-dry rotary instrument at 15 V for 15 min. The membranes were incubated with primary antibodies or antiserum, namely anti-BmFerHCH serum (1:500) and anti-β-tubulin (1:5000, TransGen). The secondary antibodies were anti-rabbit IgG conjugated to horseradish peroxidase (HRP) (1:5000, TransGen) or anti-mouse IgG conjugated to HRP (1:5000, TransGen). The HRP-DAB Chromogenic Kit (Tiangen, Beijing, China) was used to develop the protein bands. Three biological replicates were performed.

### 4.4. Reverse Transcription–Quantitative Real-Time Polymerase Chain Reaction (RT-qPCR)

Total RNA was extracted from BmN cells using the TRIzol reagent (Invitrogen, Carlsbad, CA, USA) following the manufacturer’s protocol and then reverse transcribed into complementary DNA (cDNA). The instruments and reagents used in this study for RT-qPCR were described by Cao et al. [43]. Relative quantification of target genes was calculated using the 2^−ΔΔCt^ method. Table S2 presents the primer sequences.

### 4.5. Cell Viability Assay

In order to determine the effect of the experiment processing on cell activity, the cell counting kit-8 (CCK8, Biosharp, Hefei, China) was used according to the manufacturer’s instructions. Cells were inoculated in 96-well plates at a fusion degree of about 90%. The corresponding concentration of inhibitor was added and incubated for 12 h. Then, 10 μL of CCK8 reagent was added to each well and incubated for 2 h at 27 °C. The absorbance was measured at 450 nm.

### 4.6. Construction of a Recombinant DNA Plasmid

Following Yang et al. [44], pNF-κB-Luc was generated by inserting the OpIE 2 promoter and five classic NF-κB CREs in tandem between the *Xho*I and *Hind*III restriction sites of the pGL3-basic vector. The OpIE 2 promoter was amplified by PCR using the pIZT/V5-His vector as a template. Based on the predicted information, specific primers were used to amplify the sequence (−2025, +16 nt) near the translation initiation site (ATG) of *BmFerHCH* by PCR. The PCR product was cloned into the pGL3-basic firefly luciferase reporter vector (Promega, Madison, WI, USA) using *Xho*I and *Hind*III restriction enzyme sites. The vector was named pGL3(−2025, +16). A series of vectors containing *BmFerHCH* promoter fragments were constructed using pGL3(−2025, +16) as a template, namely pGL3(−1220, +16), pGL3(−975, +16), pGL3(−540, +16), pGL3(−2025, −1199)-TATA, pGL3(−1220, −939)-TATA and pGL3(−957, −521)-TATA. TATA is the sequence that contains the core promoter region near the *BmFerHCH* translation initiation site (−243, +16). To construct the mutant vectors pGL3(−540, +16)-MutCRE1, pGL3(−540, +16)-MutCRE2, pGL3 (−540, +16)-MutCRE3 pGL3 (−957, −521)-MutCRE4, we used the Quick Mutation Plus Site Directed Mutagenesis Kit (Beyotime, Shanghai, China). All of the specific primers are shown in Table S3.

### 4.7. Analysis of NF-κB Activity

BmN cells were seeded onto 12-well cell culture plates 24 h prior to transfection. Transfection was performed using the Neofect DNA transfection reagent (Neofect, Beijing, China). The pNF-κB-Luc were co-transfected with *Renilla* luciferase reporter plasmid PAC5.1-Renilla (100 ng) after 24 h. The cells were infected with BV or treated with PDTC (an NF-κB inhibitor) for 12 h. The NF-κB-dependent luciferase activity of cell extracts from each sample was measured using Dualucif Firefly and Renilla Assay Kit (UE, Shanghai, China) according to the manufacturer’s protocol. The activity of luciferase was assessed in three independent experiments.

### 4.8. Dual Luciferase Assay

BmN cells were seeded onto 12-well cell culture plates 24 h prior to transfection. pIZ-BmRelish-act (1000 ng) or pIZ/V5-His (1000 ng) was co-transfected with the pGL3 vector containing different promoter fragments (500 ng), and the *Renilla* luciferase reference reporter plasmid PAC5.1-Renilla (100 ng). Three replicates were performed for each group. Forty-eight hours after transfection, chemiluminescence was detected by a microplate reader according to the instructions of the Dualucif Firefly and Renilla Assay Kit. Furthermore, the relative fluorescence activity was determined by dividing the firefly fluorescence signal by the sea kidney fluorescence signal. The fluorescence activity was normalised to the pGL3-basic control group.

### 4.9. Electrophoretic Mobility Shift Assay (EMSA)

To detect the binding of the RHD domain of the Relish recombinant protein to the NF-κB site in the BmFerHCH promoter, we used the Chemiluminescent EMSA Kit (Beyotime, Shanghai, China) according to the manufacturer’s instructions. The wild-type oligonucleotide sequences are probe-CRE2: AATGTAGGTTATTTCCATCTCG and probe-CRE4: AATAAGTGGGAATCTCTCCATCTCGC; each probe is labelled with biotin at the 5′ end. The mutant oligonucleotide sequences are Mut-CRE 2: AATGTACATTATCTCCATCTCG and Mut-CRE 4: AATAAGTGCCAAACACTCCATCTCGC. The reverse complementary sequences were synthesised by Sangon Biotech (Shanghai, China) and annealed to produce double-stranded DNA sequences. A competitive experiment was conducted using mutated and unlabelled wild-type probes (cold probes).

## Supplementary Materials

The following supporting information can be downloaded at: https://www.mdpi.com/article/10.3390/ijms231810380/s1.
