# Supplementary material for: Nuclear Factor Kappa B Promotes Ferritin Heavy Chain Expression in Bombyx mori in Response to B. mori Nucleopolyhedrovirus Infection
Source: Int J Mol Sci. 2022 Sep 8;23(18):10380. doi: 10.3390/ijms231810380 (PMC9499628; doi:10.3390/ijms231810380)
Supplement: Supplementary file 1 [file ijms-23-10380-s001.zip › ijms-1902526-supplementary.pdf]

Supporting Information

Table S1 The proteome data statistics of *B . mori* nuclear factor NF-kappa-B p110 subunit isoform 1

ID	Gene Description	Accession No.	P50+/P50-	BC9+/BC9-
Bm_nscaf2511 _029	nuclear factor NF-kappa-B p110 subunit isoform 1	NP_001095935.1	1.1	1.2

Table S2 List of the primers used in this study for qRT-PCR.

Name	Forward primer (5’- 3’)	Reverse primer (5’- 3’)
BmFerHCH	CTCGGACTCGTTGCTGCC	GCCACTTCCTCCTGGATCTGT
BmGAPDH	CGATTCAACATTCCAGAGCA	GAACACCATAGCAAGCACGAC
BmRelish	CGTCTGGGTCGTTGAAGAGT	GACCGAGTGTAGGTCCACG

Note: Abbreviations: BmFerHCH,*B. mori* ferritin heavy chain; BmGAPDH, *B. mori* glyceraldehyde-3-phosphate dehydrogenase; BmRelish, *B. mori* nuclear factor NF-κB P110 protein.

Table S3 List of the primers used in this study for vector construction.

Name	Primer sequence (5’- 3’)
OpIE-2-F	gggagaaca<u>ggaattc</u>CATGATGATAAACAATGTATGGTGCT
OpIE-2-R	cagtaccggaatgcc<u>aagctt</u>AACAGATGCTGTTCAACTGTGTTTAC
5×NF-κB-F	TCGAGGGGAATTTCCGGGTTTTTCCCGGGGATTTCCGGGAATTTCCGGGTTTTTCCCG
5×NF-κB-R	AATTCGGGAAAAACCCGGAAATTCCCGGAAATCCCCGGGAAAAACCCGGAAATTCCCC
pGL3(-2025)-F	gcgtgctagcccggg<u>ctcgag</u>CTAGGTACGTCGTGAGCTCA

pGL3(-1220)-F	gcgtgctagcccgggctcgagGGTTCGTTCAGTTGCTGTTAT
pGL3(-957)-F	gcgtgctagcccgggctcgagCGTCCGCACAAAGACAAC
pGL3(-540)-F	gcgtgctagcccgggctcgagTCTCGTTTTCCTTCCCCCTC
pGL3(+16)-R	cagtaccggaatgccaagcttAGAAAACAGCCCTCATCTTAA
pGL3(-1190)-R	cagtaccggaatgccaagcttATAACAGCAACTGAACGAACC
pGL3(-939)-R	cagtaccggaatgccaagcttGTTGTCTTTGTGCGGACG
pGL3(-521)-R	cagtaccggaatgccaagcttGAGGGGGAAGGAAAACGAGA
-1190TATA-F	ttcagttgctgttataagcttTCTCGCTCGCTCACATCACTC
-939TATA-F	ccgcacaaagacaacaagcttTCTCGCTCGCTCACATCACTC
-521TATA-F	ttttccttccccctcaagcttTCTCGCTCGCTCACATCAC
BmFeH-TATA-R	cagtaccggaatgccaagcttAGAAAACAGCCCTCATCTTAA
MC1-PCR-F	GCGAATAGGACTTATTTATCCGAATATCCTGCGAATTATAAGTCGCC
MC1-PCR-R	GGCGACTTATAATTCGCAGGATATTCGGATAAATAAGTCCTATTCGC
MC2-PCR-F	CTTTGTTGTGCAATGTACATTATCTCCATCTCGCTCGCTCAC
MC2-PCR-R	GTGAGCGAGCGAGATGGAGATAATGTACATTGCACAACAAAG
MC3-PCR-F	CTCGAGTCTCGTTTCCCCCCCCCCTCGTCCGAAATGTTT
MC3-PCR-R	AAACATTTCGGACGAGGGGGGGGGGAAACGAGACTCGAG
MC4-PCR-F	GGTATTAAAATCAATAAGTGCCAAACACTCCATCTCGCTATTCTCG
MC4-PCR-F	CGAGAATAGCGAGATGGAGTGTTTGGCACTTATTGATTTTAATACC
pET30a-RelishRHD-F	cccggaattcAGACCGTTTCTTCGCATAAT
pET30a-RelishRHD-R	gccgctcgagGGCGATGTACTTGAACTCCT

pIZ-Relishact-F	cccaagcttATGTCTACAACTGCCAGTGATC
pIZ-Relishact-R	cgcggatccCAGTTATCTTAGTAGCTGTTTCACT

Note: Restriction enzymes sites were underlined. Abbreviations: F, forward; R, reverse; OpIE-2,constitutive promoter for insect cell expression;TATA, The sequence that contains the core promoter region near the BmFerHCH translation initiation site (-243, +16); MC, mutant *Cis*-regulation elements; RelishRHD, RHD domain of *B. mori* nuclear factor NF-κB P105 protein; Relishact, BmRelish after excision of the ANK;
